# Complementary and Alternative Medicine in Patients With Breast Cancer: Exploratory Study of Social Network Forum Data

**DOI:** 10.2196/12536

**Published:** 2019-11-27

**Authors:** Béatrice Lognos, François Carbonnel, Isabelle Boulze Launay, Sandra Bringay, Estelle Guerdoux-Ninot, Caroline Mollevi, Pierre Senesse, Gregory Ninot

**Affiliations:** 1 Research Unit EA4556 Epsylon University of Montpellier, University Paul Valéry Montpellier France; 2 Plateforme universitaire Collaborative d’Evaluation des programmes de Prévention et de Soins de support University of Montpellier, University Paul Valéry Montpellier France; 3 University Department of General Practice University of Montpellier Montpellier France; 4 University Multiprofessional Health Center Avicenne Cabestany France; 5 Research Unit 5506, Laboratoire d'informatique, de Robotique et de Microélectronique de Montpellier, Unité Mixte de Recherche University of Montpellier Montpellier France; 6 Institut du Cancer de Montpellier Montpellier France

**Keywords:** complementary and alternative medicine (CAM), nonpharmacological interventions, cancer, social network, forum, patient

## Abstract

**Background:**

Patients and health care professionals are becoming increasingly preoccupied in complementary and alternative medicine (CAM) that can also be called nonpharmacological interventions (NPIs). In just a few years, this supportive care has gone from solutions aimed at improving the quality of life to solutions intended to reduce symptoms, supplement oncological treatments, and prevent recurrences. Digital social networks are a major vector for disseminating these practices that are not always disclosed to doctors by patients. An exploration of the content of exchanges on social networks by patients suffering from breast cancer can help to better identify the extent and diversity of these practices.

**Objective:**

This study aimed to explore the interest of patients with breast cancer in CAM from posts published in health forums and French-language social media groups.

**Methods:**

The retrospective study was based on a French database of 2 forums and 4 Facebook groups between June 3, 2006, and November 17, 2015. The extracted, anonymized, and compiled data (264,249 posts) were analyzed according to the occurrences associated with the NPI categories and NPI subcategories, their synonyms, and their related terms.

**Results:**

The results showed that patients with breast cancer use mainly physical (37.6%) and nutritional (31.3%) interventions. Herbal medicine is a subcategory that was cited frequently. However, the patients did not mention digital interventions.

**Conclusions:**

This exploratory study of the main French forums and discussion groups indicates a significant interest in CAM during and after treatments for breast cancer, with primarily physical and nutritional interventions complementing approved treatments. This study highlights the importance of accurate information (vs fake medicine), prescription and monitoring of these interventions, and the mediating role that health professionals must play in this regard.

## Introduction

### Background

Supportive care complements approved and prescribed treatments of cancer, predominantly, nonpharmacological methods called as nonpharmacological interventions (NPIs) [1] or, more imprecisely, complementary and alternative medicine (CAM). Nowadays, patients are interested in these health solutions aimed to improve quality of life, reduce symptoms, and supplement treatments. Their uses are beyond the control and/or prescription of health professionals. Supply and demand is accelerated, especially on the internet and social networks [2]. These digital platforms extend NPIs to unknown and potentially dangerous and erratic practices, such as plants from faraway countries, electronic commerce of food supplements without manufacturing control, traditional medicines, empirically selected practices, innovative startup solutions that do not have enough time or means to carry out proper clinical trials, and hidden sectarian practices. Between 30% and 40% and between 15% and 75% of the general population in the United States and Europe, respectively, use CAM [3]. The use of CAM in oncology has been increasing for the past 10 years, in particular, to reduce the side effects of chemotherapy, radiotherapy, and surgery [4]. The use varies between 18% and 83% depending on the measurement method, type of cancers, and their definition. In breast cancer, 72% of women would use it [5]. More than half would not mention them to their oncologist, contributing to the difficulty to obtain accurate frequencies of use [6,7]. Patients may argue that their lack of mention to their oncologist or general practitioner stems from their providers’ lack of question on the topic, their lack of interest, an anticipation of disapproval, or their presumed inability to help them [8]. Patients mentioned several reasons, such as the lack of information on NPIs for the management of cancers (61% of cases), the lack of question from their oncologist (60%), the thought that this does not concern the doctor (31%), the fact that their doctor might not understand the situation (20%), the fact that their doctor would disapprove (14%), and the risk that their doctor would no longer take care of them (2%) [9]. These untolds carry risks during and after cancer treatments, with NPIs potentially generating adverse effects, deleterious interactions, and noncompliance with treatments [10,11].

One way to better understand this opaque use of CAM during cancer treatment is to explore patients’ views through specialized social networks. In 2018, 3 billion people used a social network, that is, 40% of the world’s population [12,13]. About 20% of discussions on these networks are related to health [14]. Patients find a space for open dialog among peers. These platforms also allow an exchange and appropriation of medical information, in particular, to seek answers when they have not been provided by a health professional [15-17]. This need is particularly pervasive in patients with cancer treated with complex and combined therapies. About 35% of health focus groups and forums are dedicated to cancer and sharing experiences with cancer [14,18]. Approximately 50,000 new cases of breast cancer are diagnosed every year in France [19,20]. Therapies and remission rates have progressed considerably in this field, improving patients’ outcomes and minimizing treatment side effects. The major national cancer organizations and associations support the creation of patient discussion forums to promote mutual help and sharing of experiences [21]. These forums have become a valuable source of information on NPI uses.

### Objectives

The primary objective of this exploratory study was to identify and quantify CAM-related words used from posts published on health forums and social media groups of patients with cancer patients. The secondary objective was to distinguish the words among the following 5 categories of NPIs: digital, nutritional, psychological, physical, and other.

## Methods

### Design

We conducted a retrospective frequency analysis of the words used in NPIs from a database compiled from internet-based French-language forums and discussion groups of patients treated or followed for breast cancer. These specialized social networks consisted of 2 patient forums (*impatientes* and *breast cancer*), 4 Facebook discussion groups (*Breast cancer*; *Pink October 2014*; *Breast cancer, let’s talk about it*; and *Breast-cancer*), and 4 Facebook pages (*Breast cancer a merciless war*, *Breast cancer talk group*, *Breast cancer*, and *Like-breast cancer*). The French National Cancer Institute recommended these forums to patients.

The 264,249 posts published in these forums and Facebook pages (without additional information for each post, such as the number of views, comments, shares, or likes) were collected and anonymized with the agreement of the French nonprofit breast cancer patient organization. All surnames, first names, pseudonyms, and location information (eg, city, region, and facility name) were replaced with generic labels. The use of these compiled retrospective data did not require authorization from an ethics committee or a personal protection committee in accordance with French laws and regulations.

Data collection was performed at the University of Montpellier in France. All local institutional review boards approved the protocol, and the Independent Ethics Committee of Collège National des Généralistes Enseignants (Avis N° 110719118) accepted the protocol. The Ethics Committee of the College of Teaching General Practitioners (IRB No. IRB00010804) has ruled that, under the French law, the research “Complementary and Alternative Medicine in Patients with Breast Cancer: An Exploratory Study of Social Network Forums Data” was carried out in accordance with national regulations.

### Population

According to a source dated April 2018 [22], the *Impatientes* forum counted 10,576 members who have provided their birthdate (6% aged below 35 years, 18% aged 35-45 years, 32% aged 46-55 years, 28% aged 56-65 years, and 15% aged above 65 years). We used the 160,890 posts from June 3, 2006, to November 17, 2015, of 5053 participants to disseminate information about breast cancer prevention, detection, and care. The association had created a Facebook page that was followed by 720,261 people [23]. We used 16,927 posts from an unknown number of participants from 2006 to 2015.

In April 2018, 1713 people subscribed to the Facebook page *Breast cancer, a merciless war* [24]. We used the 86,432 posts from January 10, 2010, to September 28, 2015, of 1044 participants.

### Data Analysis

**Figure 1:**
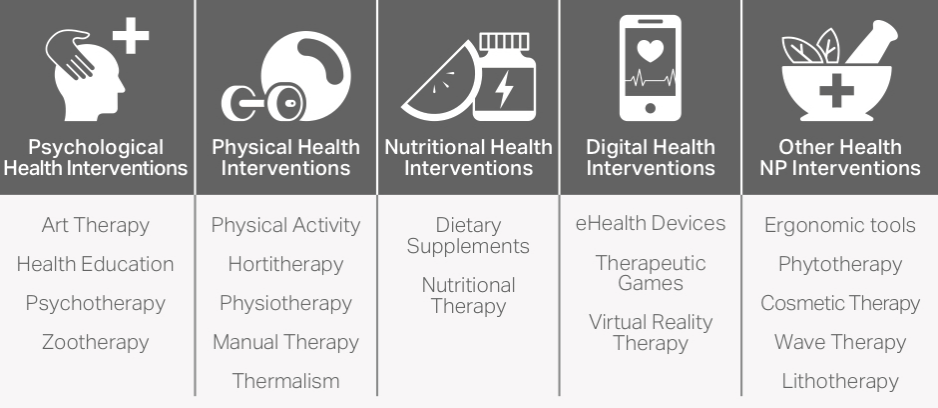
Nonpharmacological intervention ontology terms without all their related synonyms.

All NPI terms were searched in the compiled database of 264,249 posts. These queries were made from the ontology of NPIs provided by the academic and collaborative *Plateforme CEPS* (Figure 1) [1,25]. Each query considered singular/plural, abbreviations and misspellings, and words with and without dashes (eg, *non-pharmacological* and *non pharmacological*) as equivalent to the ontology’s featured word. The method consists of identifying and counting the NPI terms mentioned in the social network posts. We conducted 2 successive descriptive frequency analyses: (1) an analysis of the occurrences of NPI categories and their synonyms (Figure 1) and (2) a subcategory analysis with NPI terms, their synonyms, and their related terms (eg, ingredient, technique, method, and profession).

## Results

### Nonpharmacological Interventions’ Categories and Synonyms

Within our dataset, patients referred to an NPI category 14,185 times, 84.51% (11962/14195) from the *Impatientes* forum, 8.57% (1217/14195) from the *Breast cancer* forum, and 6.92 % (986/14195) from Facebook groups and pages (Table 1). The study population mainly referred to physical and nutritional interventions and others in similar proportions between forums and Facebook groups/pages. The term *NPI* (abbreviated or not) was rarely used by patients (20 occurrences in total), whereas the term *CAM* was never used.

**Table 1:** Occurrences of nonpharmacological intervention categories in 264,249 published posts.

Categories	Total occurrences, n^a^ (%)	*Impatientes* forum, n1^b^ (%)	*Breast cancer* forum, n2^c^ (%)	Facebook group/page, n3^d^ (%)
Physical	5197 (36.64)	4423 (36.90)	394 (32.40)	380 (38.7)
Nutritional	4437 (31.28)	3827 (31.93)	403 (33.14)	207 (21.1)
Psychological	636 (4.48)	496 (4.14)	54 (4.44)	86 (8.7)
Digital	0 (0.00)	0 (0.00)	0 (0.00)	0 (0.0)
Others	3915 (27.50)	3241 (27,03)	365 (30.02)	309 (31.5)
Total	14,185 (100.00)	11,987 (100.00)	1216 (100.00)	982 (100.0)

^a^n refers to the entire population under study.

^b^n1 refers the entire population of forum impatientes under study.

^c^n2 refers the entire population of forum breast cancer under study.

^d^n3 refers the entire population of Facebook group under study.

### Nonpharmacological Interventions’ Subcategories, Synonyms, and Related Terms

The total number of subcategories and related terms is 13,084. No mention was made in the database of terms related to digital health intervention (eg, serious game, health device, connected health tool, app, digital tool, and virtual coach). The subcategories of physical health interventions mentioned were physical activity programs (83.5%) and manual therapies (15.4%) and physiotherapy (1.1%) as shown in Table 2. The 3 physical interventions most commonly mentioned were exercise (36.4%), acupuncture (32.4%), and yoga (16.0%).

Table 3 details cited health nutrition interventions. Of the 4437 occurrences obtained, dietary supplements were most prevalent (77.9%) compared with nutritional therapies (22.1%). The most cited health nutrition interventions were vitamins (39.3%), honey (12.2%), iron (10.7%), and grapefruit (6.7%).

Table 4 presents the results of the subcategories of psychological health interventions. Psychotherapies were predominant (97.0%). The most used term was sophrology (34%).

Regarding other health interventions, the most popular was herbal medicine in forums and Facebook solutions (Table 5).

**Table 2:** Repartition of occurrences for the physical health intervention category.

Subcategories	Total occurrences (number of times the occurrence is cited)	*Impatientes* forum	*Breast cancer* forum	Facebook	Related terms^a^
Physical activity programs	3253 (+1087)^a^	2769 (+940)	228 (+84)	256 (+63)	Shiatsu, yoga, tai chi, body building, Pilates, hatha yoga, and Iyengar yoga
Horticultural therapies	0	0	0	0	—^b^
Physiotherapies	7 (+52)	5 (+39)	0 (+7)	2 (+6)	Speech therapy
Manual therapies	4 (+796)	4 (+667)	0 (+75)	0 (+54)	Acupuncture, acupressing, osteopathy, reflexology, auriculotherapy, and chiropraxy
Thermal cares	0	0	0	0	—

^a^The number of occurrences of related terms to the subcategory physical activity programs, not as a synonym (eg, exercise) but as a related term (eg, Pilates).

^b^Not applicable (no one mentioned).

**Table 3:** Repartition of occurrences for the nutritional health intervention category.

Subcategories	Total occurrence (number of times the occurrence is cited)	*Impatientes* forum	*Breast cancer* forum	Facebook	Related terms
Food supplements	488 (+2970)	413 (+2699)	53 (+150)	22 (+121)	Alpha linolenic acid, iron, gamma linolenic acid, amino acids, magnesium, minerals, niacin, ascorbic acid, palmitic acid, creatine, fish oil, biotin, calcium, bioflavin, vitamin (A, C, B, B1, B2, B3, B6, B12, D, D3, and E), multivitamin, and folic acid
Nutritional diets	2 (+977)	2 (+713)	0 (+200)	0 (+64)	Dukan diet, fasting, and micronutrition

**Table 4:** Repartition of occurrences for the psychological health intervention category.

Subcategories	Total occurrences (number of times the occurrence is cited)	*Impatientes* forum	*Breast cancer* forum	Facebook	Related terms
Health education programs	1 (+0)	1 (+0)	0 (+0)	0 (+0)	Tobacco cessation
Psychotherapies	59 (+561)	54 (+431)	2 (+51)	3 (+79)	Hypnosis, hypnotherapy, self-hypnosis, autosuggestion, sophrology, support group, and mindfulness-based stress reduction
Art therapies	2 (+1)	1 (+0)	0 (+0)	1 (+0)	Musicotherapy
Zootherapies	12 (+0)	9 (+0)	0 (+1)	3 (+0)	—^a^

^a^Not applicable.

**Table 5:** Repartition of occurrences for the other nonpharmacological intervention category.

Subcategories	Total occurrence (number of times the occurrence is cited)	*Impatientes* forum	*Breast cancer* forum	Facebook	Related terms
Cosmetic therapies	0 (+481)	0 (+289)	0 (+150)	0 (+42)	Wig and makeup
Wave therapies	12 (+1)	1 (+0)	0 (+0)	1 (+0)	Chromotherapy, light therapy, quantum medicine, electrotherapy, and magnets
Phytotherapies	83 (+2218)	79 (+1888)	1 (+146)	3 (+184)	Aloe vera, aromatherapy, belladonna, calendula, chamomile, cinnamon, milk thistle, clove, echinacea, eucalyptus, feverfew, devil’s claws, mistletoe, herbs, hops, linseed oils, essential oils, hypericum, kava, lavender, alfalfa, marijuana, peppermint, St. John’s wort, blueberry, passionflower, dandelion, elderberry, tea, red clover, valerian, cranberry, pomegranate, bitter orange, wild yam, grapefruit, cocoa, and noni
Lithotherapies	1 (+16)	1 (+15)	0 (+0)	0 (+1)	Stone
Ergonomic tools	0 (+0)	0 (+0)	0 (+0)	0 (+0)	—^a^

^a^Not applicable.

## Discussion

### Principal Findings

Our study exploring a large dataset from social networks highlights the attractiveness of NPIs for patients with breast cancer. The results from conversations in 2 forums and 4 Facebook discussion groups and pages recommended by the French National Cancer Institute indicated that 27,279 words were related to NPIs in the 264,249 posts analyzed. NPIs are clearly a topic of concern for patients with a generally similar interest in the categories between forums and Facebook sources. Patients seek information about the best use of NPIs and use these NPIs. The study supports the results of a survey conducted in 2005 on CAM by using a structured questionnaire with an average of 35.9% of a sample of 956 European patients [7]. The European study had identified 33 CAM [7], whereas this study identified 101 CAM. This figure could have been even more extensive if the patients had precisely mentioned the method rather than the profession (eg, osteopathy, acupuncture, chiropractic, speech therapy, sophrology, music therapy, light therapy, and aromatherapy) or the vector (eg, minerals and pebbles). Our study thus confirms the ability of social networks to address more deeply and broadly the NPI/CAM spectrum compared with a questionnaire survey. Indeed, the questionnaire may hinder patients from revealing their real uses and/or concerns about practices decried by some health authorities (eg, cannabis). Questionnaires restrict responses to those designed in advance by researchers. They suggest inappropriate contextual and temporal conditions for dealing with topics in depth. They may limit the representativeness of the patients interviewed, whereas the use of NPIs is known to vary according to age, gender, socioeconomic level, residence, and country [7,26]. This analysis of real-life postings makes it possible to point out original practices. If a social network reinforces personal convictions, it offers patients the opportunity to discover new practices consistent with these beliefs.

This study indicates that the words used by health professionals and researchers to describe all nonpharmacological solutions such as *NPI* or *CAM* are very rarely used by patients with breast cancer. The vocabulary used by the patients is pragmatically focused at the level of the methods of care and not at the level of their categories. One aim of digital social networks is to answer usage questions of a vast and opaque field mixing methods (eg, hatha yoga), ingredients (eg, cinnamon), disciplines (eg, physiotherapy), skills (eg, profound breath), and alternative dangerous medicines (eg, quantic medicine). Our descriptive study reveals the diversity of NPIs used by French or at least Francophone patients during breast cancer treatment and recurrence prevention. It reflects a wide range of health goals. Biologically, patients seek these nonpharmacological solutions for an improvement of the efficacy of their treatments (eg, compliance with scheduled doses of chemotherapy, prevention of cachexia, and prevention of fat gain) and a reduction of treatment side effects (eg, decreased nausea and pain or fatigue) [27]. At the psychobehavioral level, they look to reduce anxiodepressive signs (eg, self-esteem and/or body image trouble) [26,28], change health behaviors (eg, smoking cessation), and improve their quality of life.

The predominant categories are physical and nutritional interventions. These care strategies begin to be integrated into support care departments of French cancer hospitals. The physical activity subcategory is predominant and is consistent with recent mechanistic studies [29,30], meta-analyses [31,32], and authorities’ recommendations [33,34]. Although clinical trials have shown benefits of a physical activity program on quality of life and treatments side effects (eg, fatigue, depressive symptoms, and physical condition), recent studies suggest effects on the reduction of tumor growth rate [35] and the prevention of recurrence in patients aged younger than 40 years [36]. Our results testify to the capacity of social networks to convey scientific and medical messages, the subsidiary question, which our data cannot answer, being to know the modalities of practice (eg, intensity, frequency, and duration). The subcategory manual therapies are present, in particular, for practices known for their pain-relieving effect (eg, acupuncture). It should be noted that there is no vocabulary associated with spa treatments in a country known for offering many interventions reimbursed by national health insurance.

Nutritional health interventions, the second most frequently cited NPI category, have been studied by observational cohorts and pilot trials, suggesting their efficacy in curative breast cancer treatments [35,36]. The goal of maintaining a normal weight through a diet is a factor of good prognosis [35], whereas weight gain after the diagnosis of breast cancer is associated with a higher mortality rate, further increased in case of a weight gain of 10% or more [37]. If food supplements are debated in the literature [38], patients have a particular interest in them based on the frequency of citation.

A common subcategory in the other NPI category is herbal medicine. Herbal remedies are popular among cancer patients as indicated by surveys [7], despite persistent scientific doubts about their toxicity, their risk of interaction with chemotherapy, and their efficacy in reducing symptoms or acting on the tumor [39]. Another subcategory is also mentioned to a lesser extent in the category of psychological health interventions, psychotherapies. Some are beginning to be advocated in the curative pathways of patients with breast cancer to relieve anxiety symptoms, depressive symptoms, and mood disorders [40].

In contrast to our literature-based assumptions [13,15], digital health interventions were not mentioned in the studied forums and groups. Is it because of old data (before 2016) or a lack of interest of patients nevertheless sensitized to digital solutions by their participation in a social network? The results indicate that French-speaking patients with breast cancer do not care or wonder about serious games, virtual reality, and connected objects. They may not be aware of their effect on health. The generalization of oral chemotherapy with serious risks in case of misuse and the familiarization of health professionals with these solutions will undoubtedly increase the use of these digital systems (eg, pillboxes and a specific informational app). This justifies further longitudinal and prospective studies.

This study indicates the value of forums and focus groups in supporting patients during cancer and postcancer treatments [41]. At the individual level, they have a function of exchanging information, sharing experiences, recommending healthy behavior, and providing social support [21,42]. This mutual support among peers living with the same medical situation is a factor in improving quality of life [43]. Forums and discussion groups are easily and quickly accessible. They provide detailed information that is personalized, educational (*patient language*, drawings, and videos), accessible everywhere, updated, voluminous, anonymous, and free. They are a source of strategy for obtaining support to help sustain change in health behavior [44] and to think about how to collaborate with health professionals in a disease so elusive to the naked eye. This empowerment [42] facilitates the personalization of care toward integrated solutions. Patients seek to make sense of their disease to improve their health, to maximize their chances of healing/survival without recurrence/prolonging period without recurrence, to restore their femininity, and to improve their quality of life. It is legitimate for patients to seek the best solutions for treatment through all means available to them.

At the collective level, forums and discussion groups reinforce the sense of belonging to a community, access to rights, identity claims, and the desire to contribute to the improvement of care practices. As patients wonder about their care by sharing experiences through social media, they are no longer *patients* but actors in collaboration with their caregivers and community. They seek to help their neighbor. In this context, it is significant to note the development of the status of *expert patient*. Some engage in university courses to go beyond the mere experience of disease, stigma, and ostracism [45].

The study underlines the power of digital social networks to share—disseminate—recommend practices across borders of which health professionals may have little awareness. Some patients become precursors, *beta testers*, of solutions never proven or whose manufacturing quality remains to be verified. The study raises important questions about the reliability of CAM information available to patients and regulatory authorities’ responsibility for labeling, approval, and surveillance. The results sensitize health professionals and authorities to the power of forums and discussion groups to make known beneficial but also potentially dangerous solutions that currently escape the purview of regulatory and monitoring systems [46]. A recent study shows the risks of CAM in the survival of patients with cancer if they delay the establishment of prescribed cancer treatments or replace them [47]. Other studies indicate that CAM can encourage physicians to listen longer, more thoroughly, and more comprehensively to their patient [8]. More than a nebulous approach, NPIs considered as verified methods become levers of potentiation of biomedical treatments through better patient involvement (eg, adherence and maximization of placebo effect) and supplements acting on most psychosomatic symptoms (eg, nausea, sleep disorders, anxiety and depressive disorders, fatigue, and pain). The study points to a future medical challenge of accurately naming and describing NPIs to promote evidence-based practice and a future that is no longer based on empirical beliefs or advice and to have traceability of uses [48]. This will be even more central, as we see the emergence of integrated supportive care solutions where NPIs are offered as a bouquet of services by a multidisciplinary team [49,50]. Bringing health professionals together through a common vocabulary could reinforce the patient’s idea that a close-knit team is doing their utmost to treat their cancer and prevent it from recurring. In the absence of a care path validated/recommended by science and authorities, the uses are mainly based on the preferences, beliefs, and empirical practices, of which a major vector is social networks. There is an urgent need to train doctors who hold NPIs at best for simple general dietary advice and at worst for solutions with no effect on health and cancer, so that they can give clear and up-to-date scientific information to their patients who might be confused by various messages on social media.

### Limitations

Given the confidentiality required for the use of the social network data studied and the ethical framework of this study, it was impossible to know the medical characteristics (eg, type and severity of cancer, number of recurrences, treatment period, comorbidities, condition health, and risk behaviors) or personal (eg, age), social (eg, social status), and geographical (eg, France vs Francophonie) information on people who wrote a post. Moreover, it was impossible to know if posts were repeated several times by the same person, including on different social networks. Finally, the rules of confidentiality of the networks do not make it possible to affirm with certainty that all published posts emanate from patients with cancer. For example, companies can use these tools by creating virtual patients to promote their nonpharmacological products. Relatives of a sick person can also register to search for information. Impostors could also be spreading false medical information.

Although voluminous and proportional to the attractiveness of CAM, the declarative data did not distinguish interest from real use. Posting can reflect as much a request for information or a doubt as the sharing of actual use of an NPI. Qualitative approaches should complete these mass data analyses to better identify the real choices (eg, medical prescription vs autoprescription) and context-specific uses. It is essential to know whether these practices are used in a complementary or alternative way to approved and prescribed cancer treatments [46].

Analyses were performed on data compiled between 2006 and 2015. With more data and a longer period of time, it would be interesting to study the chronology of the vocabularies used by patients about NPIs to identify potential *fashion* effects. [51].

### Conclusions

The exploratory study of breast cancer patient forums and Facebook discussion groups raises important questions about the reliability of CAM information available to patients and regulatory authorities’ responsibility for labeling, approval, and surveillance. Health professionals and authorities need to be sensitized to the power of forums and discussion groups to make known beneficial but also potentially dangerous solutions that currently escape the purview of regulatory and monitoring systems as mentioned by a recent study.

## Abbreviations

CAM: complementary and alternative medicine
NPI: nonpharmacological intervention
